# Supplementary material for: Inter-System Variability of Eight Different Handheld Ultrasound (HHUS) Devices—A Prospective Comparison of B-Scan Quality and Clinical Significance in Intensive Care
Source: Diagnostics (Basel). 2023 Dec 26;14(1):54. doi: 10.3390/diagnostics14010054 (PMC10795594; doi:10.3390/diagnostics14010054)
Supplement: Supplementary file 1 [file diagnostics-14-00054-s001.zip › diagnostics-2790257-supplementary.pdf]

Supplementary Material

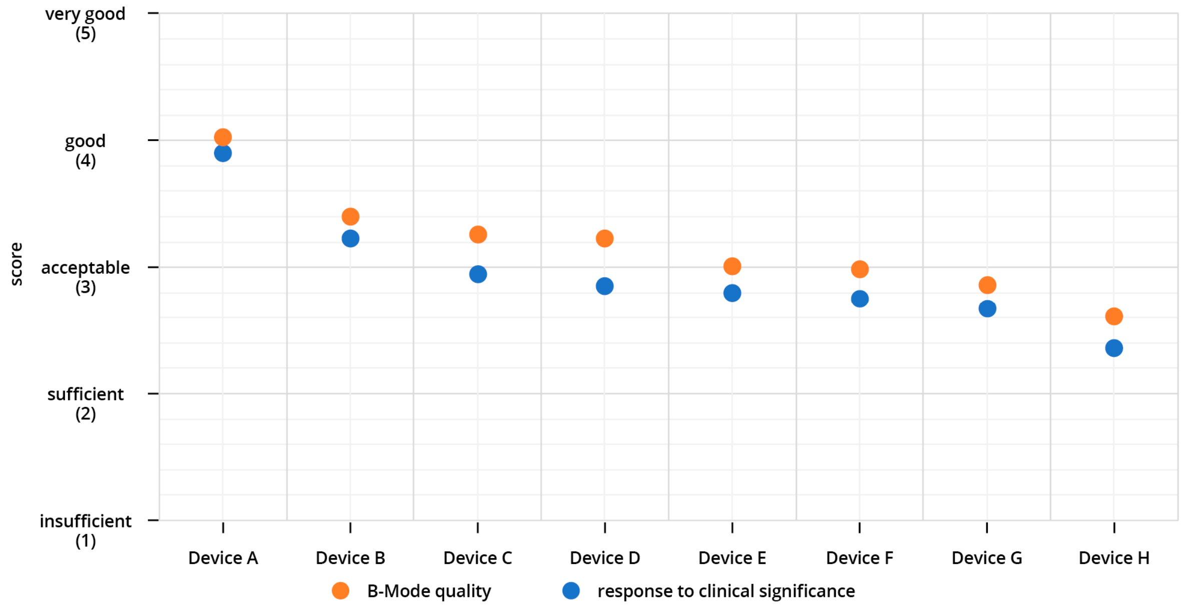

(**a**)

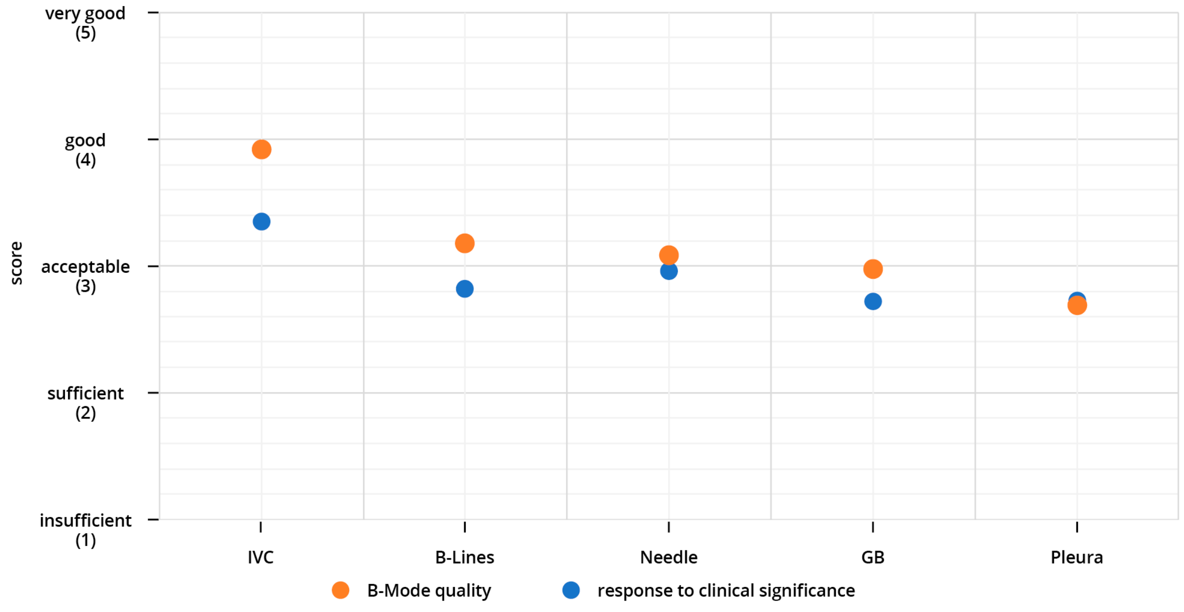

(**b**)

Figure S1. Comparison of the scores obtained from both evaluations for each device used (**a**) and for each sonographic question (**b**).

Table S1. Results of the evaluated B-scan quality per evaluator (a) and Results of the evaluated clinical significance per evaluator (b).

(a) B-Scan Quality

EvaluatorNo.	Device A	Device B	Device C	Device D	Device E	Device F	Device G	Device H
**1**	4.2	3.4	3.2	2.6	3.2	3.6	3.4	3.0
**2**	3.8	3.2	2.6	2.0	2.8	2.8	2.2	2.4
**3**	4.0	3.0	3.0	3.2	2.8	3.0	3.0	2.4
**4**	3.6	3.2	3.0	3.0	3.0	2.6	2.8	2.2
**5**	3.8	3.4	3.4	2.8	2.4	2.8	2.4	2.2
**6**	4.4	3.2	3.4	3.4	3.0	2.8	3.6	2.8
**7**	3.8	3.0	3.2	3.0	2.6	3.2	2.8	2.8
**8**	3.8	3.2	2.8	3.2	2.8	2.0	2.8	2.4
**9**	3.4	3.0	3.2	2.6	2.8	3.0	1.8	2.4
**10**	3.8	3.6	3.2	3.2	2.8	2.8	3.0	2.6
**11**	4.2	3.8	3.0	3.0	2.8	3.2	2.8	2.6
**12**	3.6	3.0	2.8	2.6	2.8	3.2	2.4	2.2
**13**	3.6	2.0	2.4	2.2	2.4	2.0	2.2	1.8
**14**	4.4	3.0	3.0	3.0	2.8	3.2	3.0	2.6
**15**	3.6	2.2	2.4	2.2	2.0	2.0	2.0	1.8
**16**	4.4	2.8	2.6	2.6	3.0	1.8	2.6	1.8

(b) Clinical Significance

EvaluatorNo.	Device A	Device B	Device C	Device D	Device E	Device F	Device G	Device H
**1**	4.4	3.6	4.0	4.0	3.2	3.4	3.2	3.2
**2**	3.6	3.4	2.4	2.2	3.0	2.6	2.4	2.0
**3**	4.2	3.4	3.2	3.6	3.0	3.2	3.0	2.8
**4**	3.8	3.8	3.0	3.2	3.2	3.2	3.0	2.8
**5**	3.8	3.4	3.4	3.0	2.4	2.8	2.6	2.6
**6**	4.8	3.6	3.8	2.6	3.6	2.4	3.6	2.6
**7**	3.6	3.2	3.6	3.8	3.6	3.6	3.0	3.2
**8**	4.0	3.8	3.0	3.8	2.8	3.2	3.4	2.6
**9**	3.2	3.6	3.6	2.8	3.0	2.8	2.6	2.4
**10**	3.4	3.6	3.2	3.4	2.8	2.8	3.2	2.4
**11**	4.4	4.4	3.8	3.8	3.2	4.0	3.0	2.8
**12**	3.8	3.0	3.0	2.8	2.8	3.2	2.2	2.2
**13**	4.2	2.6	3.0	2.8	3.0	2.2	2.6	2.4
**14**	4.8	3.2	3.4	3.4	3.4	3.6	2.8	3.2
**15**	4.0	2.8	2.8	2.4	2.6	2.4	2.6	2.4
**16**	4.4	3.0	3.0	3.0	2.6	2.4	2.6	2.2

Table S2. P-values in the comparison of the B-scan quality of the individual HHUS devices (a); P-values in the comparison of the clinical significance of the individual HHUS devices (b); p < 0.01 marked in red, p < 0.05 and > 0.01 marked in green.

p-value
B-scan Quality

	Device A	Device B	Device C	Device D	Device E	Device F	Device G
Device B	<0.001						
Device C	<0.001	0.5					
Device D	<0.001	0.18	0.45				
Device E	<0.001	0.13	0.36	0.8			
Device F	<0.001	0.06	0.2	0.65	0.76		
Device G	<0.001	0.006	0.03	0.28	0.36	0.47	
Device H	<0.001	<0.001	<0.001	0.004	0.008	0.013	0.023

p-value Clinical Significance

	Device A	Device B	Device C	Device D	Device E	Device F	Device G
Device B	<0.001						
Device C	<0.001	0.55					
Device D	<0.001	0.46	0.88				
Device E	<0.001	0.07	0.22	0.31			
Device F	<0.001	0.045	0.16	0.24	0.87		
Device G	<0.001	0.001	0.008	0.03	0.24	0.32	
Device H	<0.001	<0.001	<0.001	0.002	0.03	0.04	0.15

Table S3. P-values in the comparison of the B-scan quality of the different sonographic questions (a); P-values in the comparison of the clinical significance of the different sonographic questions (b); p < 0.01 marked in red, p < 0.05 and > 0.01 marked in green.

**p-value** B-scan Quality				
	IVC	B-Lines	Needletracking	Peural Effusion
B-Lines	0.06			
Needletracking	<0.001	0.28		
Pleural Effusion	<0.001	0.038	0.35	
Gallbladder	<0.001	0.05	0.43	0.98
**p-value** Clinical Significance				
	IVC	B-Lines	Needletracking	Peural Effusion
B-Lines	<0.001			
Needletracking	<0.001	0.63		
Pleural Effusion	<0.001	0.17	0.42	
Gallbladder	<0.001	<0.001	0.009	0.039

Table S4. P-values (Anova analysis) in the comparison of the B-scan quality and the clinical significance of the different HHUS devices (marked in green: $p < 0.05$)

**B-scan Quality**	Device A	Device B	Device C	Device D	Device E	Device F	Device G
**Clinical Significance**	Device A	Device B	Device C	Device D	Device E	Device F	Device G
**p-value**	0.12	**0.02**	**0.03**	**0.03**	0.17	0.10	0.23

Table S5. P-values (Anova analysis) in the comparison of the B-scan quality and the clinical significance of the different sonographic questions (marked in green: $p < 0.05$; marked in red: $p < 0.01$)

**B-scan Quality**	IVC	B-Lines	Needle Tracking	Pleural Effusion	Gallbladder
**Clinical Significance**	IVC	B-Lines	Needle Tracking	Pleural Effusion	Gallbladder
**p-value**	**< 0.001**	0.12	0.07	**0.012**	0.83
